# King Edward's Memorial Homes for Nurses

**Published:** 1921-05-21

**Authors:** 


### King Edward's Memorial Homes for Nurses.

The promoters of the King Edward Memorial Home for Retired Nurses in Glasgow, who have been appealing for £25,000 to establish such a home on a sound financial basis, are fortunate in having received the gift of Hazelwood House, Dumbreck, Glasgow, for this purpose. The house and grounds were given to the Red Cross during the war and it is hoped to adapt it for the use of aged and retired nurses by November 1922. Already over £5,000 has been raised, chiefly by means of sales of work at the Royal, Western, Greenock, and Victoria Infirmaries, and the Oakbank Hospital. The well-planned movement for identifying the memory of King Edward with the establishment of homes in different parts of the country for retired nurses has dropped a little out of the public eye, but has already succeeded so far in its aims that three homes—one at Clapham, one at Holland Park, and one in Edinburgh—are in existence. They are for nurses not able to do active work, but yet able to look after themselves, keep their rooms in order, and attend meals. There is no age limit, and several nurses between the ages of seventy and ninety-five are in residence. There is great need for one in Glasgow, and here the movement owes much to the exertions of Miss Melrose, whose recent retirement from the matronship of the Royal Infirmary has caused widespread regret.

Adequate nursing for such as need it is indispensable to the proper management of such homes, but the more the residents are left at liberty to order their lives in their own way, and meet their own responsibilities, the less it is likely to be required. Nurses least of all women are likely to fall into the semi-childish dependence on others which used to be thought proper for all over sixty. Their vigour and independence of outlook should not be sapped by fussy regulations.

